# Human Skin Keratinocytes on Sustained TGF-β Stimulation Reveal Partial EMT Features and Weaken Growth Arrest Responses

**DOI:** 10.3390/cells9010255

**Published:** 2020-01-20

**Authors:** Sergio Liarte, Ángel Bernabé-García, Francisco J. Nicolás

**Affiliations:** Laboratorio de Regeneración, Oncología Molecular y TGF-β, IMIB-Arrixaca, El Palmar, 30120 Murcia, Spain; sdll1@um.es (S.L.); a.bernabegarcia@gmail.com (Á.B.-G.)

**Keywords:** TGF-β, keratinocytes, chronic wounds, smads, EMT, cell proliferation

## Abstract

Defects in wound closure can be related to the failure of keratinocytes to re-epithelize. Potential mechanisms driving this impairment comprise unbalanced cytokine signaling, including Transforming Growth Factor-β (TFG-β). Although the etiologies of chronic wound development are known, the relevant molecular events are poorly understood. This lack of insight is a consequence of ethical issues, which limit the available evidence to humans. In this work, we have used an in vitro model validated for the study of epidermal physiology and function, the HaCaT cells to provide a description of the impact of sustained exposure to TGF-β. Long term TGF-β1 treatment led to evident changes, HaCaT cells became spindle-shaped and increased in size. This phenotype change involved conformational re-arrangements for actin filaments and E-Cadherin cell-adhesion structures. Surprisingly, the signs of consolidated epithelial-to-mesenchymal transition were absent. At the molecular level, modified gene expression and altered protein contents were found. Non-canonical TGF-β pathway elements did not show relevant changes. However, R-Smads experienced alterations best characterized by decreased Smad3 levels. Functionally, HaCaT cells exposed to TGF-β1 for long periods showed cell-cycle arrest. Yet, the strength of this restraint weakens the longer the treatment, as revealed when challenged by pro-mitogenic factors. The proposed setting might offer a useful framework for future research on the mechanisms driving wound chronification.

## 1. Introduction

Keratinocytes constitute the outermost layer of the skin, the epidermis, whose main function is creating a physical barrier against environmental insult. Skin wounds imply the loss of integrity of the epithelial barrier, even though the initial disruption may affect deeper tissue layers [1]. For the purpose of successful restoration of the epidermal continuity, keratinocytes undergo sequential proliferation and migration. This is coordinated by a myriad of growth factors and cytokines (e.g., EGF, VEGF, TGF-β, PDEGF, and IL1) produced by a variety of cell types. These factors regulate the functions of all cell types involved and their presence evolves in space and time with the overlapping phases, which define the healing process: (i) inflammatory, (ii) proliferative, and (iii) remodeling [2]. Skin lesions that fail to orderly proceed through these phases are termed chronic wounds. Pathologically, the clearest indicator of such a condition is the inability of the epidermis to complete epithelialization. This feature is common to venous ulcers, pressure ulcers, diabetic ulcers, and massive traumatic wounds [3]. Accumulated evidence indicates that resident cells in chronic wounds might develop abnormal responses to cytokines, including aberrant reaction to Transforming Growth Factor-β1 (TGF-β1) [3,4,5,6].

Amongst growth factors and cytokines influencing wound healing, TGF-β would promote the broadest spectrum of effects [7]. In mammals, TGF-β seems crucial for both skin homeostasis and repair and its aberrant regulation has been linked to cutaneous pathologies like psoriasis [8] and fibrosis [9]. The TGF-β family comprises three isoforms (TGF-β1, -β2 and -β3) which, play a pivotal role by regulating responses in every cell type involved in wound healing [10]. For instance, in acute wounds, the release of TGF-β1 by platelets promotes fibroblast migration, and the proliferation and secretion of extracellular matrix (ECM) components. In contrast, TGF-β opposes mitogenic activity of growth factors like EGF in keratinocytes [6]. All TGF-β isoforms are highly conserved, sharing a strong identity and very similar three-dimensional structures. It is noteworthy that all the TGF-β isoforms bind to the same two universally expressed transmembrane receptors (TβRI; TβRII) with similar affinity, thus, being interchangeable for in vitro studies [11]. Upon ligand binding, TβRI and TβRII arrange into heterotetramers, which induce serine/threonine kinase activity of TβRI in receptor-specific Smads (R-Smads). Smad proteins, TGF-β signaling intracellular transducers, are divided into three groups: R-Smads (Smad2 and Smad3), a common Smad (Co-Smad4), and inhibitory Smads (I-Smad6 and 7). Phosphorylated R-Smads associate with Co-Smad4 and translocate to the nucleus [12]. These complexes interact with additional factors on target genes, effectively modulating transcription with relevant repercussions for both physiological state and disease [6]. Additionally, accumulated evidence shows the contribution of Smad-independent signaling to TGF-β effects, involving relevant intracellular mechanism including Ras/MAPK/Erk, PI3K/Akt, TAK1-p38/JNK, Par6 or Rho GTPases pathways, with effects on cell polarity, growth and apoptosis [13,14,15]. Consequently, both canonical Smad-dependent and non-canonical TGF-β signaling are involved in regulating cutaneous wound healing [16].

Research on strategies targeting TGF-β signaling to improve the regeneration in skin pathological conditions, including chronic wounds, shows confronted evidence [10,17]. The findings obtained in rat and rabbit models indicated the beneficial effects of TGF-β administration; however, research on the use of TGF-β treatment on human skin failed to meet the expectations [7,18]. Moreover, evidence obtained in vitro using human keratinocytes fails to reproduce the positive effects of TGF-β addition [19,20]. More importantly, there is mixed evidence in the current knowledge of TGF-β dynamics in human wounds. For acute skin lesions, TGF-β levels increase sharply and decrease over time during the clotting and healing process [5,18]. However, this does not hold true in all instances. Available evidence in humans indicates that, while reduced levels dominate at the dermis compartment, elevated TGF-β levels are sustained over time in the epidermis of chronic wounds [21,22,23]. In this line, data on transgenic mice overexpressing TGF-β1 in keratinocytes show histological characteristics, which are similar with those of the epidermis of chronic wounds, suggesting that excessive and prolonged TGF-β expression at the wound site does not benefit healing [24]. This differentiated pattern of elevated TGF-β in the epidermis, which seems to constitute a hallmark of chronic ulcers, confronts the extended notion that low TGF-β expression dominates in chronic wounds [25].

Regardless of the state of underlying dermal structures, skin wounds cannot be considered closed if they lack complete epithelialization. Competent keratinocyte proliferation and migration constitute a necessary requisite for successful re-epithelization and wound closure [26]. Precisely, during wound healing, TGF-β signaling has been proposed as a key factor in regulating epithelial-to-mesenchymal transition (EMT) for keratinocytes. However, there is lack of evidence on the potential effects of persistent exposure to TGF-β in chronic wounds. To address that issue, we propose a cellular model based on the spontaneously immortalized epithelial cells HaCaT derived from human skin. These cells are characterized for extended passage lifespan and exhibit cell-to-cell contact inhibition of proliferation, even though they are not of malignant origin. They also retain typical keratinization after confluence disruption, providing a standardized system to analyze wound progression through time, while enabling easy experimental replication [27,28,29]. The sustained exposition of HaCaT cells to recombinant TGF-β1 provides a meaningful tool to understand cell behavior and the molecular events leading to the impaired epithelization found in chronic wounds. Changes brought about by TGF-β chronic exposure are discussed at the cellular and molecular levels.

## 2. Materials and Methods

### 2.1. Cells Culture Conditions and Treatments

HaCaT cells [27], obtained from ATCC, were cultured in full medium (FM) comprising Dulbecco’s modified eagle high-glucose medium (Biowest, Nuaillé, France), completed with 10% FBS (Thermo Fisher Scientific, Waltham, MA USA), 1000 units/mL penicillin, 1000 μg/mL streptomycin (Sigma-Aldrich, St Louis, MO, USA) and 1% l-glutamine (Biowest, Nuaillé, France), at 37 °C in a 7.5% CO_2_ controlled atmosphere. Where indicated, cells were changed to serum deprived medium (SS). TGF-β1 (PeproTech, Rocky Hill, NJ, USA) was inoculated at 2 ng/mL. For samples maintained continuously in the absence or presence of TGF-β, culture medium was refreshed every 24 h and new TGF-β1, if indicated, was inoculated. Epidermal Growth Factor (EGF; Sigma-Aldrich, St Louis, MO, USA) was supplemented at 10 ng/mL. Supplementation with FBS was achieved by introducing fresh FBS into SS samples up to a final 10% concentration, or 10% extra supplementation making up to 20% final when introduced to cells already in FM.

### 2.2. Cell Morphology and Islet Cell Density

Reference images from HaCaT cells subjected to different treatments were obtained by using an inverted phase-contrast microscope incorporating a CCD camera and applying matching picture settings for magnification and digital resolution. To determine islet cellular density values, a total of fifty cell islets per condition were characterized by using ImageJ Fiji distribution and applying area drawing and cell counting functions [30].

### 2.3. Quantitative PCR

Total RNA was extracted from sub-confluent cell cultures (65%) using the RNeasy-mini system (Qiagen, Venlo, The Nederlands). Typically, 1 μg of RNA from independent samples was retro-transcribed using iScript reagents (Bio-Rad, Hercules, CA, USA). Resulting cDNA was used for quantitative PCR (qPCR) implementing the SYBR premix ex Taq kit (Takara Bio Europe/Clontech, Saint-Germain-en-Laye, France) according to manufacturer’s instructions. For each mRNA, gene expression levels were normalized to the Glyceraldehyde 3-phosphate dehydrogenase (GAPDH) content of each sample by applying the comparative Cq method (2^−∆∆Cq^). Primers used are detailed in Table S1. Replicates from three independent experiments were quantified. Analyzed data represent mean ± SEM.

### 2.4. Western Blot

HaCaT cells were seeded in FM to reach 50% confluence on day two in 6 cm diameter plates. At this point, culture medium was substituted for SS and cells were maintained in the presence or absence of TGF-β1 for indicated times. Cells were lysed using lysis buffer: 20 mM TRIS pH 7.5, 150 mM NaCl, 1 mM EDTA, 1.2 mM MgCl_2_, 0.5% Nonidet P-40, 1 mM DTT, 25 mM NaF, and 25 mM β-glycerolphosphate (all from Sigma-Aldrich, St Louis, MO, USA) supplemented with phosphatase inhibitors (I and II) and protease inhibitors (all from Sigma-Aldrich, St Louis, MO, USA). Protein extracts were quantified using the Bradford method. Normalized total protein loadings were analyzed by SDS-PAGE followed by western blotting (WB) using the appropriate antibodies: anti-phospho-Smad2, anti-phospho-Smad3 (Cell Signaling Technology, Danvers, MA, USA), anti-Smad2/3 (Beckton Dickinson, Franklin Lakes, NJ, USA), anti-Smad3 (Abcam, Cambridge, UK), anti-phospho-ERK1/2, anti-pan-ERK, anti-phospho-JNK1/2, anti-phospho-c-Jun (Cell Signaling Technology, Danvers, MA, USA), anti-c-Jun, anti-Vimentin, anti-E-Cadherin (Santa Cruz Biotechnology, Heidelberg, Germany). Secondary antibodies were anti-rabbit IgG horseradish peroxidase linked F(ab’)2 I fragment from donkey (GE Healthcare, GE, Little Chalfont, United Kingdom) and horseradish peroxidase linked rat anti-mouse IgG1 (Beckton Dickinson, Franklin Lakes, NJ, USA). WBs were revealed using horseradish peroxidase substrate (ECL; GE Healthcare, GE, Little Chalfont, United Kingdom). Images were obtained with a Chemidoc XRS^®^ (Bio-Rad, Hercules, CA, USA).

### 2.5. Immunocytochemistry and Image Analysis

HaCaT cells were grown in FM on round glass coverslips. Upon achieving 65% approximate confluency, indicated culture conditions and TGF-β1 inoculation were applied. At determined times, coverslips with attached cells were extracted and fixed using formaldehyde (Applichem GmbH, Darmstadt, Germany) at 4% in PBS. Then, cells were permeabilized in 0.3% Triton X-100 (Sigma-Aldrich, St Louis, MO, USA) in PBS. Unspecific staining blocking containing 0.3% bovine serum albumin (Santa Cruz Biotechnology, Heidelberg, Germany), 10% FBS (Thermo Fisher Scientific, Waltham, MA USA), 0.1% Triton X-100 (Sigma-Aldrich, St Louis, MO, USA) in PBS and 5% skimmed milk supplementation (Beckton Dickinson, Franklin Lakes, NJ, USA) was applied. Immunostaining was performed using a vehicle similar to blocking solution but devoid of skimmed milk. Primary antibodies were anti-Smad2/3 (Beckton Dickinson, Franklin Lakes, NJ, USA), anti-Smad2 (Cell Signaling Technology, Danvers, MA, USA), anti-Smad3 (Abcam, Cambridge, UK), anti-panCytokeratin, anti-E-Cadherin, anti-Vimentin, anti-Catenin-β (Santa Cruz Biotechnology, Heidelberg, Germany) or anti-alpha-SMA (Sigma-Aldrich, St Louis, MO, USA). Appropriate fluorescent-conjugated secondary antibodies were used together with Alexa fluor 594-conjugated phalloidin (Molecular Probes, Thermo Fisher Scientific), where indicated, and Hoechst 33258 (Fluka, Biochemika, Sigma-Aldrich, St Louis, MO, USA) to reveal actin, and nuclei, respectively. Appropriate positive controls were performed allowing for the consideration of cases in which negative signal was retrieved. Images were taken with a confocal microscope (LSM 510 META from Zeiss, Jena, Germany).

### 2.6. Cell Cycle and Apoptosis Analysis

The cells were seeded in FM on 6 cm culture plates and grown to 35% confluence, then specific conditions and treatments were applied. For pre-conditioning studies, the cells were transferred to SS conditions. Control samples remained in SS for the corresponding times while assay samples were exposed to TGF-β1 for 24 h or 48 h. After pre-conditioning, post-treatments were performed by introducing FBS or EGF alone or together with fresh TGF-β1 inoculation for 24 h. Cells were harvested by detachment with trypsin/EDTA (Biowest, Nuaillé, France). Cell counts and viability determinations were routinely performed using trypan blue dye (Sigma-Aldrich, St Louis, MO, USA) and a TC10 automated cell counter (Bio-Rad, Hercules, CA, USA). For cell cycle analysis, samples from detached cells were centrifuged and the resulting pellet was immediately fixed with ice cold 70% ethanol. The cells were then washed three consecutive times with cold PBS (Thermo Fisher Scientific, Waltham, MA USA) in order to remove ethanol and they were finally stained for cell cycle assessment. Briefly, the cells were treated with a solution of 20 μg/mL Ribonuclease A and 40 μg/mL propidium iodide (all from Sigma-Aldrich, St Louis, MO, USA) in PBS. To assess apoptosis, the cells were analyzed for the surface presence of Annexin V and DNA content following manufacturer instructions (Beckton Dickinson, Franklin Lakes, NJ, USA). For both procedures, cells were analyzed through flow cytometry using a FACS Calibur 1 (Beckton Dickinson, Franklin Lakes, NJ, USA).

### 2.7. Statistics

Data from colony density and qPCR were analyzed with analysis of variance (ANOVA) applying Bonferroni’s multiple comparison correction and using the Prism’s Graph Pad Data Analysis software. *p*-values lower than 0.05 were considered to be statistically significant. In figure legends, asterisks denote statistically significant differences between conditions and treatments (* *p* < 0.05, ** *p* < 0.005, *** *p* < 0.001 and **** *p* < 0.0001).

## 3. Results

### 3.1. Long-Term TGF-β1 Exposure Alters the Conformation of HaCaT Cells

**Figure 1:**
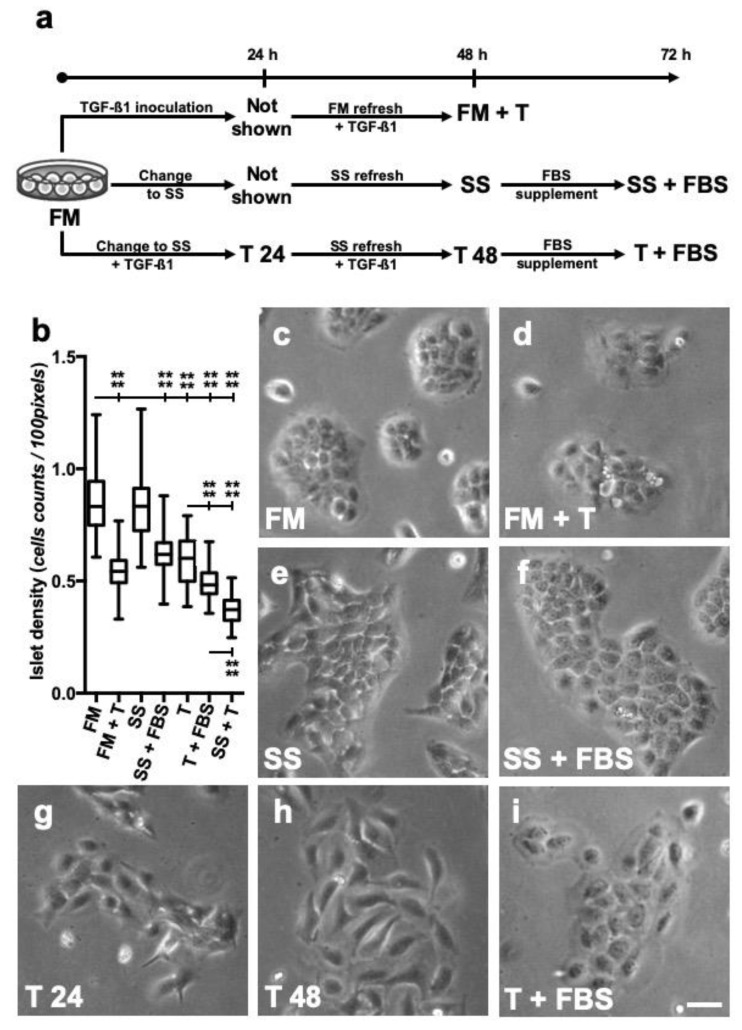
Continuous TGF-β1 treatment causes unique phenotype changes in HaCaT cultured in serum starved conditions. (**a**) HaCaT keratinocytes were maintained in different culture conditions, either in the presence (FM) or absence (SS) of FBS, with, or without, TGF-β1 (T) as indicated in the diagram. (**b**) Islet density is defined as the cell counts of a coherent group of cells divided by the surface covered by it. Shown boxes represent mean ± SEM, whiskers are indicative of outlying data. Data from three different experiments are shown. Asterisks denote statistically significant differences between conditions and treatments (**** *p* < 0.0001). Detail of islet cell morphology for each condition assayed: (**c**) cells in full medium (FM); (**d**) cells grown in full medium and inoculated with TGF-β1 [+ T]; (**e**) cells maintained in serum starvation conditions (SS); (**f**) cells maintained in SS conditions for 48 h and then supplemented with fetal bovine serum (FBS); (**g**) cells maintained 24 h in SS with TGF-β1 (T); (**h**) cells maintained in SS with T for 48 h; (**i**) cells maintained in SS with T for 48 h and then supplemented FBS. A set of representative pictures from at least three different experiments is shown. Scale bar: 50 µm.

Spontaneously immortalized human keratinocytes (HaCaT) were put in culture in the presence of continuous TGF-β1 for more than 48 h (Figure 1a). To avoid interference from factors carried in serum, samples that were exposed to TGF-β were subjected to serum starvation, by changing to serum deprived medium (SS). HaCaT cells evolved into different morphologies in response to continuous TGF-β1 stimulation and depending on the culture conditions used. Changes correlated well with variations in cells size, evidenced as islet cell density, which is expressed as the number of cells belonging to a coherent group, divided by the surface covered (Figure 1b). A close look at the cells growing in full medium (FM) revealed the usual groups of packed polygonal-shaped cells (Figure 1c). This conformation was somewhat retained when cells were simultaneously exposed to TGF-β1 up to 48 h; however, bigger round-shaped cells with signs suggestive of cellular protrusion were observed at the margins (Figure 1d). Moreover, islet cell density was reduced (Figure 1b). Serum starvation (SS) conditions are often used in assessing HaCaT responses to growth factors and cytokines. HaCaT cells maintained 48 h in SS conditions conformed aggregated groups with cell density similar to that of cells cultured in FM; however, cells at the margins of these groups tended to present with elongated shapes (Figure 1e). Re-introduction of FBS for 24 h resulted in an apparent recovery of the initial phenotype, however, cell density somewhat decreased (Figure 1f). In the case of cultures using SS conditions and exposed simultaneously to TGF-β1, cells treated just for 24 h showed changes such as elongated cell shapes and reduced density groups (Figure 1g). After 48 h TGF-β1 treatment in SS conditions, cell changes further evolved into a spindle-shaped-like phenotype with scarce signs of protrusions and developing in lower density groups (Figure 1h). In that case, FBS re-introduction resulted in a great increase in size, with cells showing rounded shape and apparent signs of protrusion (Figure 1i).

These observations suggested that HaCaT keratinocytes experience unique phenotype changes when exposed to TGF-β1 and SS conditions for longer periods.

### 3.2. HaCaT Cells Continuously Exposed to TGF-β1 Exhibit a Distinct Gene Expression Profile

**Figure 2:**
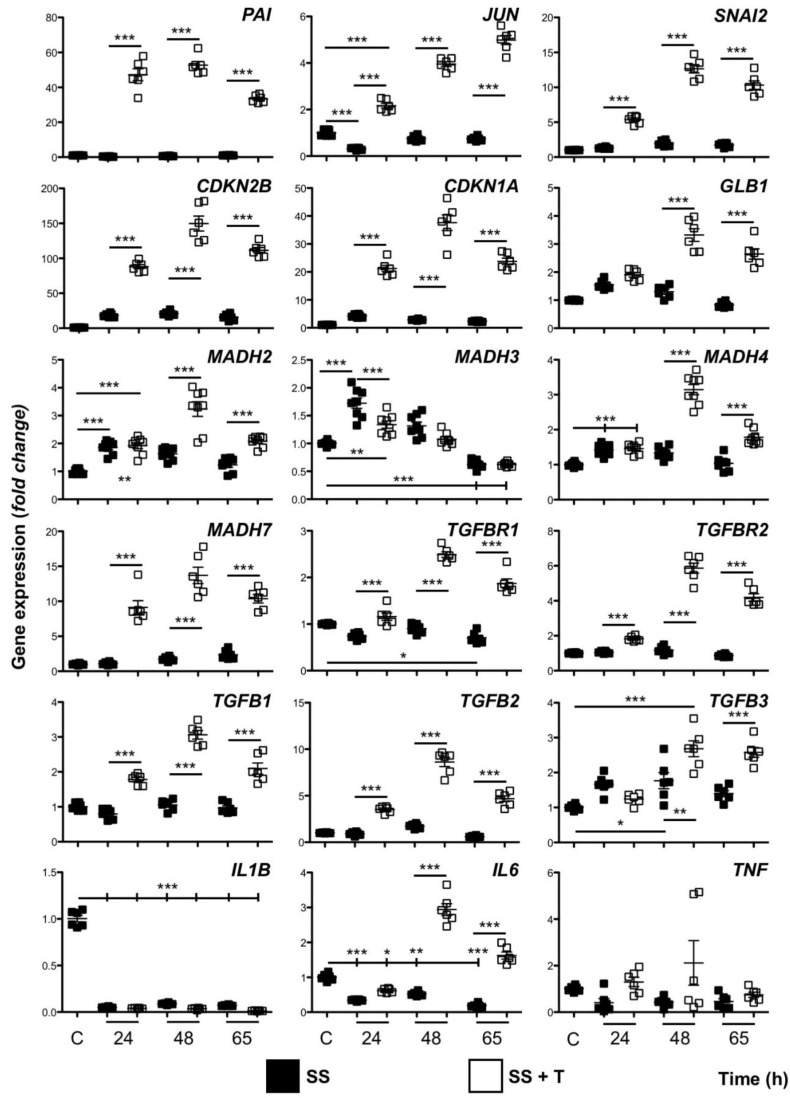
Gene transcription evolves throughout extended treatments with TGF-β1. HaCaT cells maintained in serum-deprived conditions were exposed to 2 ng/mL TGF-β1 for the indicated periods of time. Culture medium and TGF-β was refreshed every 24 h. Cells growing in full medium containing fetal bovine serum were used as reference controls C. Expression level data are represented as fold change from control samples. Replicates from three independent experiments were quantified by qPCR. Shown data represent mean ± SEM. Asterisks denote statistically significant differences (* *p* < 0.05, ** *p* < 0.005 and *** *p* < 0.001). SS: serum starvation; + T: TGF-β1 exposure.

Most cellular responses to TGF-β can be attributed to the regulation of gene transcription. For the majority of the markers studied, quantitative mRNA analysis of HaCaT cells, maintained in SS conditions, did not show relevant differences when compared to cells cultured in FM. However, SS cultures, simultaneously exposed to TGF-β1 for up to 65 h, showed generalized changes with different patterns of gene expression. *JUN* (c-Jun) and *PAI-1* are regarded as early response genes to TGF-β1 [29,31,32]. Analysis of their mRNA levels showed patent induction sustained over time, increasing progressively for *JUN,* while some decline was found at the end of the study for *PAI-1* (Figure 2). Genes *CDKN1A* (p21) and *CDKN2B* (p15), both involved in growth arrest, exhibited a strong response through time, which was characterized by an evident initial increase at 24 h, followed by peak expression levels at 48 h, and some moderation after 65 h (Figure 2). Remarkably, several other genes studied, which code for TGF-β signaling pathway proteins, behaved in a similar fashion. This was the case for *MADH2* (Smad2), *MADH4* (Smad4), *MADH7* (Smad7), *TGFB1* (TGF-β1), and *TGFB2* (TGF-β2), although the variation in expression levels was little when compared to that of non-TGF-β1 treated control samples. Interestingly, *TGFB3* (TGF-β3) levels began to increase after 48 h exposure to TGF-β1 as well, though no reduction of induction was seen (Figure 2). *TGFBR1* (TβRI), *TGFBR2* (TβRII), and *GLB1* (β-Galactosidase) genes also showed induction and partial decline, although increased levels became evident only after 48 h of treatment. The same applied to *SNAI2*, a transcription factor involved in EMT. However, *MADH3* (Smad3) pattern differed from the changes described so far. Its expression level increased after 24 h, but progressively declined later regardless of the treatment, reaching levels slightly below the levels of FM samples (Figure 2). Regarding genes related to inflammation, *IL1B*, *IL6,* and *TNF*, SS conditions seemed to promote downregulation to some extent. Significant induction was recorded only for IL6 and cells exposed to TGF-β1, but only after longer exposures (Figure 2).

The results described above provide a detailed description of the evolution of the transcriptomic effects that longer TGF-β1 exposure combined to SS conditions had on HaCaT keratinocytes. This suggests that regulation of elements contributing to TGF-β signaling at different levels might play a role.

### 3.3. Serum-Deprived Hacat Cells Exposed in the Long Term to TGF-Β1 Do not Experience Complete Epithelial-to-Mesenchymal Transition

**Figure 3:**
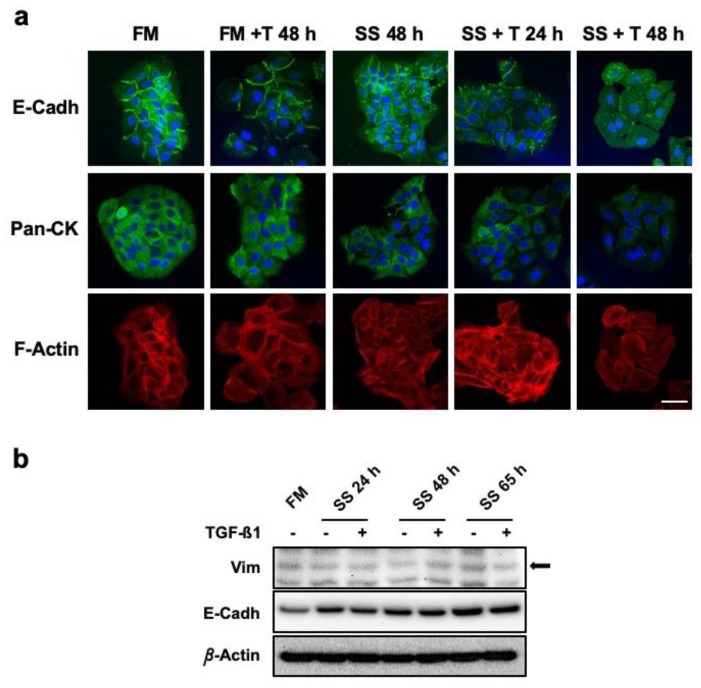
HaCaT keratinocytes do not experience EMT in response to continuous TGF-β1 exposure. (**a**) Confocal microscopy studies on the expression and localization of structural markers E-Cadherin (E-Cadh), Citokeratins (Pan-Ck), and actin filaments (F-Actin) with, or without, TGF-β1. Scale bar: 50 µm. **(b)** Protein levels of Vimentin (Vim) and E-Cadherin (E-Cadh) were assessed by Western Blot. Total protein extracts from HaCaT cells cultured for the indicated times in SS conditions, with or without TGF-β1, were compared to control sample (FM). β-Actin was used as protein loading control. Arrow indicates the correct identity of protein bands. Representative images of at least three independent experiments are shown. FM: full medium; SS: serum starvation; + T24 h, + T48 h: time (hours) of continuous TGF-β1 stimulation.

Epithelial cells acquiring fibroblastoid or spindle cell shape is considered a hallmark of EMT. This phenomenon, usually linked to cancer progression, is known to involve TGF-β signaling. In that sense, here we reported the induction of *SNAI2* by TGF-β1. In order to verify whether cell changes observed were constitutive of an EMT, we studied related relevant markers at the sub-cellular level. In SS conditions, TGF-β1 continuous treatment promoted extensive rearrangement of cell adhesion structures. This was defined by decreasing E-Cadherin immune-labelling associated with the periphery of cells together with increased detection in the cytoplasm (Figure 3a). The Keratin content analyzed by means of a pan-cytokeratin antibody revealed an evident and progressive reduction only in samples exposed to TGF-β1 in SS conditions. Cells in FM conditions showed extensive development of F-Actin intermediate filaments, especially for cells exposed to TGF-β1. F-Actin architecture for cells in SS conditions was very similar to those in FM, however, cells in SS and exposed to TGF-β1 presented with progressively longer structures sustaining cell changes in both shape and size (Figure 3a). Interestingly, the study of Vimentin, an EMT structural signature, showed no evident marking for cells in FM nor in SS regardless of TGF-β1 presence (Figure S1). In order to corroborate these findings, we studied total protein expression of E-Cadherin and Vimentin. Strikingly, Vimentin showed low basal protein levels with no relevant changes in response to TGF-β1. For the case of E-Cadherin, a slight increase in protein content was recorded, which affected all samples and treatments over time (Figure 3b).

These data about HaCaT cells confirmed the occurrence of significant alterations affecting the cell structure and adhesion components of keratinocytes in response to sustained TGF-β1 exposure in the absence of serum. Interestingly, our observations did not support the development of a complete EMT.

### 3.4. HaCaT Cells Long Term Cultured in SS And TGF-β1 Show Down-Regulation of Smad3 Protein Levels and Present with Altered Signalling

**Figure 4:**
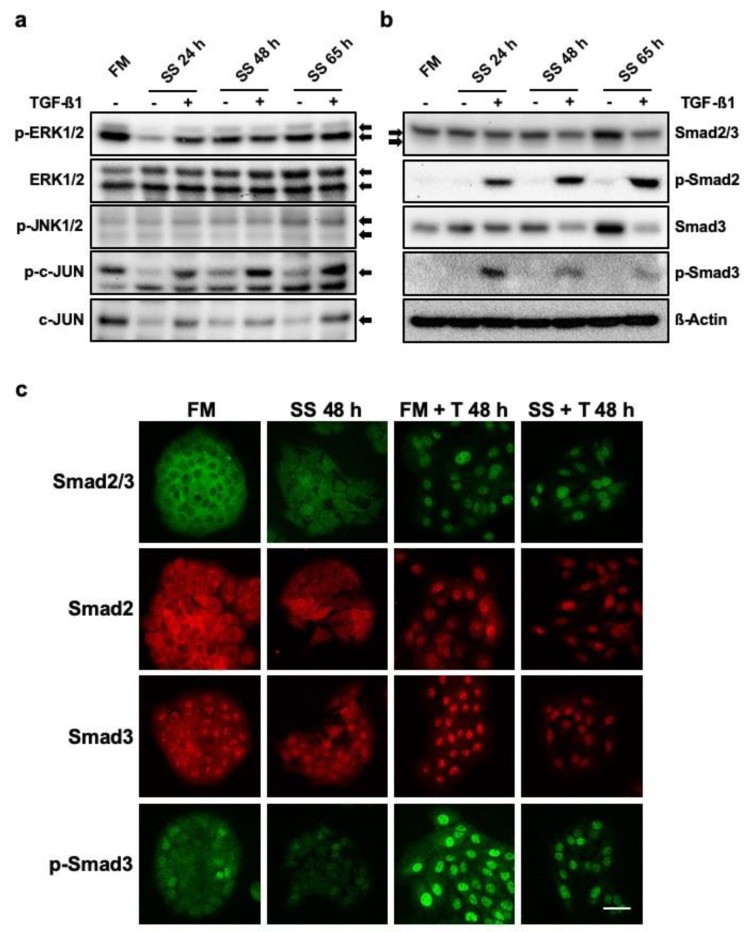
Continued exposure to TGF-β1 in HaCaT cells affects the expression of proteins involved in its transduction pathway. The expression and activation status of the indicated proteins involved in either canonical; (**a**) or non-canonical (**b**) TGF-β pathways were assessed by means of Western Blot. Normalized total protein extracts from HaCaT cells cultured for the indicated times in SS conditions, with, or without, TGF-β1, were compared with control samples from cells growing in FM. β-Actin was used as protein loading control. Arrows indicate the correct identity of protein bands. p-: phosphorylated protein form. (**c**) Changes in the protein expression and localization of receptor regulated Smads in response to continued TGF-β1 exposure. Representative confocal microscopy images of at least three independent experiments are shown. Scale bar: 50 µm. FM: full medium; SS: serum starvation; + T48: time (hours) of continuous TGF-β1 exposure.

The results of gene expression upon continuous TGF-β stimulation allowed us to infer that regulation of elements participating in TGF-β signaling might be a mechanism contributing to the changes observed. We checked the status of both the canonical and the non-canonical TGF-β signaling pathways. Factors belonging to the MAP-Kinases family are commonly studied for the analysis of the non-canonical signaling pathway. The study of phosphorylated ERK1 and 2 isoforms revealed clear activation after 24 h TGF-β1 exposure, however, longer exposures showed similar levels of ERK1/2 activation both for TGF-β treated and SS controls. Although no conspicuous changes were recorded for total ERK1/2 levels, a slight increase in protein content was appreciated over time (Figure 4a). Apparently, activated JNK levels also seemed to steadily increase with no remarkable difference between SS and TGF-β1 samples. In contrast, both c-Jun activation and expression showed clear differences, with weaker labeling in SS samples in contrast with a stronger signal just for TGF-β1 stimulated samples, whose levels were close to the final time levels recorded for FM controls (Figure 4a). TGF-β1 canonical signaling was analyzed using antibodies recognizing R-Smads. An antibody detecting both Smad2 and Smad3 allowed for the detection of progressive induction of both R-Smads in SS samples. On the contrary, for the case of TGF-β1 treated SS samples, an apparent reduction could be noticed. When studying Smad2 activation, the expected phosphorylation pattern was found. At all study times, only samples exposed to TGF-β1 showed signs of phosphorylation with no apparent decline (Figure 4b). In contrast, Smad3 showed an intense effect on its protein levels, while for SS samples it appeared to accumulate over time, a progressive reduction was registered for TGF-β1 treated samples. In this regard, phosphorylated Smad3 levels also dropped sharply with time for TGF-β1 exposed samples (Figure 4b).

The study of subcellular distribution of proteins allow for better characterizing signaling functionality. Cells cultured in either FM or SS conditions showed the ability of TGF-β1 to promote characteristic R-Smad nuclear translocation. It is noteworthy that an antibody binding total Smad2 and Smad3 fractions retrieved similar labeling for samples exposed for 48 h to TGF-β1, regardless of the culture conditions (Figure 4c). The signal intensity was also similar when an antibody specifically recognizing Smad2 was applied (Figure 4c). Interestingly, although Smad3 increased in the nucleus, labelling intensity was apparently decreased for the case of samples exposed in the long term to TGF-β1 (Figure 4c). The use of an antibody labeling phosphorylated Smad3 confirmed a much lower content of activated Smad3 over time (Figure 4c).

These data offer valuable hints on the effects that sustained exposure to TGF-β1 have on HaCaT keratinocytes at the protein level, confirming alterations in the expression of elements involved in TGF-β both canonical and non-canonical signaling, with special relevance for Smad3.

### 3.5. HaCaT Cells Exposed in the Long Term to TGF-β1 Show Altered Cell-Cycle Arrest Responses

**Figure 5:**
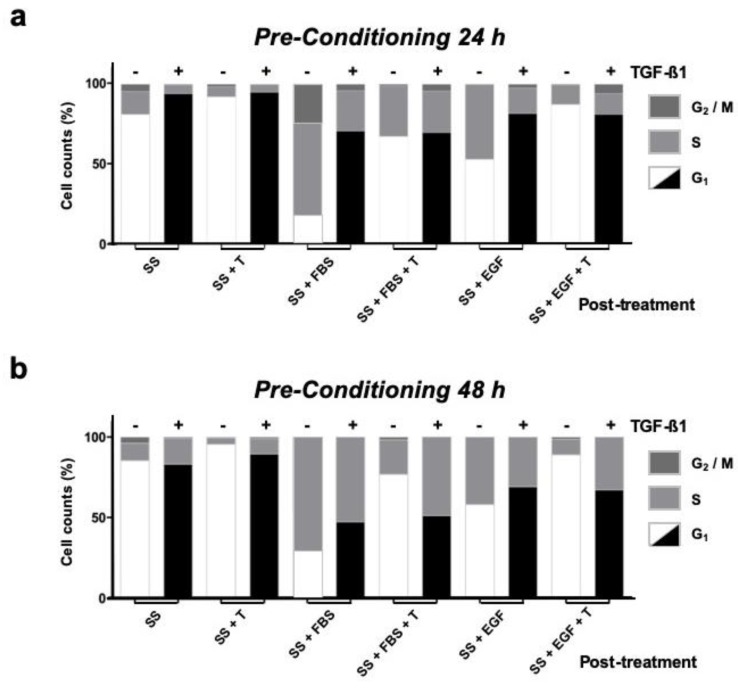
HaCaT cells under continuous TGF-β1 treatment show impaired TGF-β1 restraint with serum or EGF stimuli. Keratinocytes in serum deprived medium were conditioned by continued exposure to TGF-β1 [+] for either 24 h (**a**) or 48 h (**b**). Untreated cells (-)were used as reference controls. Response to post-treatment stimulus was determined by flow cytometry. Black G_1_ columns represent cells maintained in continuous serum deprived conditions with TGF-β1; white G_1_ columns represent control cells maintained in serum deprived conditions without TGF-β1. SS: Serum starvation medium; + T: TGF-β1 inoculation; + FBS: Foetal bovine serum supplementation; EGF: Epidermal growth factor inoculation. Representative data from at least three independent experiments are shown.

TGF-β is known to trigger powerful G1 cell-cycle arrest on non-transformed epithelial cell lines. Flow cytometry analysis of cells cultured in FM conditions showed expected responses to overnight TGF-β1 exposure, such as increased G1 fractions, even with extra FBS supplementation (Figure S2). Cells maintained in SS, both for 24 h and 48 h, showed fractions dominated by G1 cells, which increased with longer exposure. Moreover, for cells in SS, overnight inoculation of TGF-β1 further increased the G1 fraction percentage (Figure S2). The responses of cells maintained in SS and continuously exposed to TGF-β1 for either 24 h or 48 h (pre-conditioning) were also studied, along with parallel SS controls. For that purpose, cells were challenged to continue with cell cycle by means of either FBS supplementation or EGF inoculation. Cells kept for 24 h with TGF-β1 showed no effective signs of change or adaptation of their response, and neither FBS nor EGF were capable of producing significant reductions in G1 fractions, especially when compared with those levels registered for cells only maintained in SS (Figure 5a). Remarkably, when fresh TGF-β1 was inoculated together with FBS or EGF in SS samples, counts where similar to those recorded for TGF-β1 pre-conditioned cells (Figure 5a). For 48 h SS controls, either FBS addition or EGF inoculation still produced decreased G1 fractions (Figure 5b). To our surprise, cells pre-conditioned with TGF-β1 for 48 h experienced greater G1 reductions in response to FBS or EGF challenge compared with the G1 fractions registered by cells pre-conditioned for 24 h (Figure 5b). Strikingly, for 48 h pre-conditioned cells, fresh TGF-β1 inoculation together with the serum or EGF challenge also failed to maintain G1 fractions. In contrast, the expected arrest responses were observed for cells kept for the same time in SS conditions and then exposed to TGF-β1 together with either, mitogenic challenge (Figure 5b). 

Together, these observations offer useful insight on the evolution of HaCaT cell-cycle responses when exposed to TGF-β1 for long periods. Our results indicate that the changes observed at the molecular level might yield an increased sensitivity to pro-mitotic signals, or perhaps, reduced strength of the cell cycle arrest response induced by TGF-β. 

## 4. Discussion

Active TGF-β isoforms are thought to dictate cellular activities that are crucial for coordinated and successful wound repair [16,33,34]. These responses greatly depend on targeted cells being distinct at dermal or epidermal tissue compartments. For endothelial cells and fibroblasts, TGF-β develops chemotactic and pro-mitotic roles, which are essential for the conformation of granulation tissue during the inflammatory and proliferative phases of wound healing. Additionally, for fibroblasts, TGF-β mediates proper ECM replacement during the remodeling phase [35,36]. In epidermal keratinocytes, well-known responses to TGF-β include cell cycle regulation, changes in the expression of keratins and cell architecture, as well as the promotion of cell migration [20,33,34,37,38]. The evolution of these cell responses would be established on distinct spatial and temporal expression patterns, thereby, dictating TGF-β availability. In acute wound healing, TGF-β1 is provided by the abundant release from platelets; later, on the aggregated contributions by endothelial cells, fibroblasts, and keratinocytes themselves [39]. Yet, the available evidence indicates that elevated TGF-β levels sustain over time in the epidermis of chronic wounds [21,22,23,25]. Interestingly, fibroblasts isolated from chronic wounds exhibit unresponsiveness to TGF-β1 [4]. In contrast, it is unclear whether epidermal keratinocytes develop changes in relation to TGF-β1 signaling in the context of chronic wounds. In vitro keratinocyte models are mostly hindered by inherent limitations imposed by primary cultures. This restricts knowledge on the role that precise growth factors and cytokines, like TGF-β, play in specific contexts, such as wound healing, because SS conditions are often required to avoid noise emerging from factors carried in serum, and these are usually not tolerated by primary culture models. In an effort to circumvent these limitations, we used HaCaT cells in our study. They are a valued research model for studying keratinocyte physiology and wound healing, capable of enduring SS conditions for periods of several days with no apparent irreversible change [28]. However, to our knowledge, no specific setting has addressed the effects of long exposure to TGF-β on these cells in such conditions yet.

The HaCaT cells continuously exposed to TGF-β1 retained hallmarks of functional TGF-β signaling, e.g., growth arrest response, R-Smad phosphorylation, and the induction of early response genes [20,32,40,41]. Also, in response to sustained TGF-β1, the cells evolved into different morphologies depending on the absence or presence of serum. These divergent phenotypes might emerge from the integration of inputs through both canonical and non-canonical TGF-β pathways. Regarding the non-canonical signaling factors studied, we found changes affecting their contents and activation status. TGF-β’s ability to promote activation of c-Jun N-terminal Kinases, JNK1 and JNK2, has been demonstrated in several cell models [13]. Notwithstanding this, our results indicate that the slight increase observed for JNK activation might respond only to SS conditions. In this regard, JNK activation has been related to environmental stress, including ionizing radiation, heat, oxidative stress, or DNA damage, thus, being implicated in the development of the apoptotic responses related to disease [42]. Noteworthy, JNKs are also known to crosstalk with the MEK/ERK pathway [42]. Our results indicate that in this model, induction and activation of ERK1/2 might be a consequence of sustained SS conditions, with the exception of shorter exposure to TGF-β1. ERK1/2 activation in relation to serum deprivation has been reported in the past for both mesenchymal and epithelial cell lines, and it is regarded as a mechanism that modulates the apoptosis and promotes cell survival in restrictive conditions [43,44]. It is worth noting that our assessment of apoptosis did not show meaningful differences between either FM controls or SS samples, with, or without, TGFβ stimulation (Table S2). Altogether, it could be questioned whether transitioning to SS conditions might constitute enough stress to promote JNK and MEK/ERK activity. It might be argued that this model, using SS conditions, has limitations for assessing responses through non-canonical TGF-β signaling. However, we report meaningful phosphorylation of the transcription factor c-Jun only for samples exposed to TGF-β1. This might indicate that precise integration of signals accurately directing JNK and MEK/ERK activities are at play. Interestingly, conditional c-Jun mutations on the epidermis are known to delay wound closure [45]. As c-Jun performs a key role as an ultimate element integrating signaling from different growth factors [42]. We believe that, despite the disadvantages, our results would support the validity of the non-canonical component in this setting.

Regarding canonical signaling, the sustained induction of *JUN*, later verified by means of WB, confirmed its functionality regardless of continued TGF-β1 exposure. However, the majority of the markers studied shared an mRNA expression pattern, which was characterized by progressive induction followed by partial decline, but retaining increased levels. To our surprise, that pattern held true for most markers directly involved in the TGF-β signaling pathway, thus suggesting the development of regulation dynamics in response to continued TGF-β1 exposure. For TβRs, turnover is known to depend on endocytosis. After activation, internalized heterodimers can be recycled back to the membrane or undergo degradation [46]. Interestingly, increased degradation has been associated with enhanced Smad7 levels [47]. Thus, the induction of *MADH7* in our setting could be consequential to accommodate persistent TβRs activity. However, as our data show, *TGFBR1* and *TGFBR2* expression are induced rather than repressed. These results contrast with what has been found in fibroblasts from chronic wounds, which show decreased *TGFBR2* expression resulting in functional impairment of TGF-β signaling [4]. Similar mRNA expression patterns were registered for *MADH2* and *MADH4*. Among the genes analyzed that coded for factors involved in TGF-β signaling, *MADH3* was the only one which did not follow the common pattern, showing significant mRNA downregulation in response to persistent signaling. It is noteworthy that, although detectable total Smad3 protein levels were observed, activated Smad3 signal decreased greatly along with continued TGF-β1 exposure. Notwithstanding, WB results suggested slightly decreased protein levels for Smad2 as well, although no significant variation was recorded for its activation. These observations are interesting because R-Smads play different roles and variations in their contents are known to condition their effects [29,48]. Tight regulation of Smad2 has been reported as necessary for adequate homeostasis of the epidermis, its overexpression being linked to the hyperproliferation of basal keratinocytes in transgenic mice [49]. On the other hand, in mice, the deletion of Smad3 resulted in keratinocyte proliferation and accelerated re-epithelialization [50]. Considering the foregoing, detrimental effects could be expected from Smad3 overexpression. The results previously obtained in our lab might support that notion, as in HaCaT cells overexpression of Smad3 partially hinders the induction of migration by in vitro treatment with Amniotic Membrane [51]. In sum, these observations suggest that epidermis keratinocytes, chronically exposed to TGF-β1, would maintain the functionality of the TGF-β canonical pathway. Yet, regulatory feedback may finely and timely control of R-Smads activity, especially for Smad3, potentially shaping functional cell responses.

TGF-β is considered to be a main driver of EMT, a process implying dramatic changes which provide epithelial cells with unusual capacities usually related to tumor transformation, such as the ability to migrate through basement membranes and resistance to apoptosis [52,53]. EMT responses are regarded as crucial for proper keratinocyte migration during wound healing, yet precise knowledge to this regard is scarce [26]. *SNAI2*, coding for Slug, belongs to the Snail family of zinc finger transcription factors, and plays a key role in EMT development. *SNAI2* induction is found in keratinocytes at the margins of healing wounds in both, mouse and human in vivo and in vitro models [54,55]. Here, we report induction of *SNAI2* in response to TGF-β1. Yet, its expression pattern shows decline when exposed for longer periods, which suggests that it might also be subjected to regulatory feedback. To that extent, Smad3 has been suggested to mediate in TGF-β-induced EMT [56]. Moreover, some evidence indicates that crosstalk between Smad3 and JNK occurs during these responses [57]. In that sense, for HaCaT keratinocytes, concurrent MAP-Kinase activity has been reported to potentiate TGF-β1 responses beyond growth arrest and into EMT by means of Ras activation [20]. This is in line with our observations on incipient Vimentin filaments, suggesting that the phenotype observed on HaCaT cells in FM and TGF-β1 might correspond with a full EMT estate. In contrast, the results on the cells continuously exposed to TGF-β1 in SS conditions suggest that these cells might not experience complete EMT. This is supported by the cytoskeleton reorganization and in special by the altered distribution of E-Cadherin; more importantly, by the absence of Vimentin up-regulation nor formation of filaments. These observations are in line with current debate on the existence of the partial and intermediate EMT states which are relevant in regeneration processes, including wound healing [58], thus, indicating that our findings on the exposure of TGF-β1 and the exacerbation of phenotype differences imply potential advantages to keratinocyte in the context of wound healing.

Seemingly in opposition to EMT, TGF-β signaling is known to promote sharp cell cycle arrest in non-transformed epithelial cell lines. This response, termed cytostasis, involves downregulation of *Myc* and the consequential induction of *CDKN1A* and *CDKN2B*, coding for p21, and p15, respectively [59]. These proteins inhibit cyclin-dependent kinases CDK2 and CDK4, thus halting cell cycle progression [60]. Our results demonstrate how cytostasis is sustained throughout long-term TGF-β1 exposure. That holds essentially true for samples exposed for intermediate times, as they develop responses similar to those of cells exposed de novo. However, in cells exposed for longer, the increased proportion of cells undergoing S/G2/M upon FBS or EGF challenge showed weakened arrest. Existing literature indicates that TGF-β-driven cytostasis could be modulated by altering the Smad3 to Smad2 ratio. While, overexpressing Smad2 has no effect on several epithelial cell lines, including HaCaT, its depletion by means of RNA interference results in sharper arrest responses. In contrast, Smad3 depletion seems to be enough to interfere with growth arrest [48,61]. In that sense, keratinocytes from areas next to the edges of chronic wounds show signs of abnormal differentiation, including strata thickening (hyperkeratosis) and the presence of nuclei in the corneal layer (parakeratosis) [62]. Interestingly, these features suggest reactive proliferation rather than cytostasis. As indicated, our data here evidenced changes in the Smad3 to Smad2 ratio, which might sustain the observed evolution of arrest responses when mitogenic challenge was applied. In sum, as TGF-β levels on the epidermis of chronic wounds appears to be increased rather than reduced, the evidence detailed here suggest that under persistent TGF-β signaling, the evolution observed for the R-Smads ratio might constitute a key mechanism explaining the abnormal behavior of the epidermis cells around chronic wounds.

Our results also showed induction of *GLB1*. Its product, β-Galactosidase, is regarded as an established senescence marker. Cellular senescence constitutes a concomitant element in chronic wounds, as keratinocytes from aged humans show β-Galactosidase activity four times higher than cells from younger donors [63,64]. Additionally, senescent keratinocytes in chronic wounds are characterized by p21 up-regulation [65]. In this sense, our results rule out that senescence responses might derive from the conditions of our setting, as neither *GLB1* nor *CDKN1A* showed significant expression variation with SS conditions. The same applies to Plasminogen Activator Inhibitor-1 (*PAI-1*), which is regarded as another indicator of senescence by some authors [66]. In wounds, PAI-1 facilitates re-epithelialization by protecting ECM components from proteolytic degradation [67]. Excessive expression and activation of ECM proteolytic enzymes is characteristic of chronic wounds [62]. Here we showed *PAI-1* expression fluctuating in response to lengthy TGF-β1 treatment, experiencing final partial decline. Interestingly, heteromeric complexes that only incorporate Smad3 control *PAI-1* gene expression [68]. Moreover, Smad3 has already been related to senescent keratinocytes. Its absence has been linked to reduced GLB1 expression and β-galactosidase activity, delaying senescence in a mouse multistage skin carcinogenesis model. Moreover, Smad3 overexpression in this model resulted in impaired keratinocyte proliferation [69]. All this suggest that Smad3 plays a key role in mediating senescence responses in keratinocytes, potentially offering new research options for the role of TGF-β in chronic wounds. Indeed, it should be noted that cell senescence is also characteristic of inflammatory contexts [65]. In this regard, senescent cells remain far from static, engaging in the modulation of their environment by developing a secretome particularly rich in inflammatory cytokines and chemokines [70]. For the case of cytokines, during wound healing, the expression of all TGF-β isoforms is known to occur in response to TGF-β1 released by platelets [39,71]. Our results, that showed gene induction for all the TGF-β isoforms in HaCaT cell under sustained TGF-β1 exposure, are in line with those observations. Interestingly, evidence obtained in mesenchymal models, fibroblasts and chondrocytes, suggests that TGF-β’s ability to induce the expression of its own isoforms might be Smad3 dependent [72,73]. On the other hand, TGF-β1 is known for triggering the release of early pro-inflammatory response factors, including Il-1β, Il-6 and TNF-α [7]. Our results showed patent *IL1B* downregulation, probably related to the SS culture conditions. However, the expression pattern of *IL6*, which is similar to that of other aforementioned genes, suggests connections with TGF-β signaling and its modulation. It is worth noting that IL-6 is considered to play a key role in sustaining senescence as part of the associated secretome [74,75]. In the epidermis, IL-6 and its receptor are constitutively expressed in the basal keratinocyte layer [76], and are thought to contribute to wound healing by promoting migration, proliferation, and keratin reorganization [77,78]. Interestingly, several cellular models, not including keratinocytes, demonstrated how the AP-1 complex binds to *IL6* promoter [79,80,81]. The AP-1 family includes c-Jun, whose results in this work were already discussed. Intriguingly, increased IL-6 levels have been linked to EMT responses in several epithelial cell models in the context of a senescent secretome [82,83,84,85,86]. Few reports describe the ability of TGF-β to promote IL-6 induction: In Tenon’s capsule fibroblasts, IL-6 autocrine signaling, in response to TGF-β, was linked to alpha-Smooth Muscle Actin expression, myofibroblast transdifferentiating and scarring [87]; a similar loop has been reported in prostate epithelial carcinoma cell lines, for which a synergistic mechanism involving Smad2, NF-kappaB and MAP-Kinases was proposed [88]. As a curiosity, a mechanism described in human renal proximal tubular epithelial cells linked IL-6 signaling with increased TβR recycling and enhanced TGF-β responses [89]. Considering the fact that TGF-β is regarded as a main driver of wound scarring and fibrosis [90,91], senescence being a common characteristic of cells within these conditions, our data on HaCaT keratinocytes suggest that intricate crosstalk involving IL-6 and TGF-β autocrine loops might contribute to building senescent responses found in chronic wounds.

The etiologies that lead to the development of chronic wounds are reasonably understood. In most cases of venous stasis ulcers, chronic pressure ulcers, diabetic ulcers, or massive traumatic wounds, their inability to heal relates to defects on the keratinocyte’s capability to re-epithelialize. This has been commonly associated with persistent inflammation; however, the molecular events driving closure impairment are poorly known. In this regard, the role of continuous TGF-β signaling in the epidermis of chronic wounds has been largely overlook. A framework, based on elevated levels of TGF-β in the epidermis, sets an unexplored paradigm for chronic wound healing research; yet, access to biopsy samples from patients is usually vetoed to avoid co-morbid complications. Among the available keratinocyte models, HaCaT cells provide a resourceful study tool to investigate cytokine effects. Here we report conditions for the analysis of HaCaT responses in a TGF-β congested environment. The fact that HaCaT cells offer different responses to long-term TGF-β exposure, under prolonged SS conditions, should be regarded as a strength of the proposed experimental setting, as impaired perfusion is expected in chronic wounds as a consequence of inflammation and blood-vessels disruption. This is a key element in assessing diabetic patients, who are especially characterized by disturbed angiogenesis and lymphangiogenesis [92]. The evolution observed for cell cycle arrest responses might support the idea that the altered R-Smads balance, emerging from continuous TGF-β exposure, might significantly impact keratinocyte proliferative behavior during wound healing, thus, potentially challenging the role historically ascribed for TGF-β in chronic wounds. Finally, we find the idea that keratinocytes exposed to relevant levels of TGF-β for long periods may develop meaningful autocrine loops incorporating TGF-β and IL-6 crosstalk very interesting. Further works on this issue might allow for the verification of the existence of such crosstalk. If confirmed, establishing the specific roles for non-canonical and Smad2/Smad3-dependent canonical pathways may offer new possibilities for incorporating related senescence markers into future wound assessment. The data discussed in this work has the potential to provide guidance to future research on the mechanisms driving wound chronification and injury-related skin senescence.

## Supplementary Materials

The following are available online at https://www.mdpi.com/2073-4409/9/1/255/s1, **Table S1**: List of primers used for real time PCR studies.; **Table S2.** Apoptosis assessment. HaCaT cells were maintained either in full medium (FM) or serum deprived conditions (SS), in the absence or presence of TGF-β1 for the indicated time. Data represent percentage of combined early and late apoptotic cell fractions from three independent experiments ± SEM; **Figure S1**: Continuous TGF-β1 stimulation does not induce Vimentin filaments in HaCaT cells.; **Figure S2**: Acute TGF-β1 treatment induces cell cycle arrest to HaCaT keratinocytes.
