# Supplementary material for: Human Skin Keratinocytes on Sustained TGF-β Stimulation Reveal Partial EMT Features and Weaken Growth Arrest Responses
Source: Cells. 2020 Jan 20;9(1):255. doi: 10.3390/cells9010255 (PMC7017124; doi:10.3390/cells9010255)
Supplement: Supplementary file 1 [file cells-09-00255-s001.pdf]

Table S1. List of primers used for real time PCR.

Primer Name	Primer Sequence 5′to 3′
GAPDH-Fwd	ACCACAGTCCATGCCATCAC
GAPDH-Rev	TCCACCACCCTGTTGCTGTA
PAI 1 II-Fwd	GACATCCTGGAACTGCCCTA
PAI 1 II-Rev	GGTCATGTTGCCTTTCCAGT
c-JUN-Fwd	GGAAACGACCTTCTATGACGATGCCC
c-JUN-Rev	GGCGCGCACGAAGCCCTCGGCGAACC
SNAI2-Fwd	GATGCCGCGCTCCTTCCTGGTC
SNAI2-Rev	GCTGCTTATGTTTGGCCAGCC
CDKN2B-Fwd	ATGCGCGAGGAGAACAAG
CDKN2B-Rev	CTCCCGAAACGGTTGACTC
CDKN1A-Fwd	ATGTCAGAACCGGCTGGGGATG
CDKN1A-Rev	GGGCTTCCTCTTGGAGAAGATC
GLB1	Proprietary sequence (Sigma KiCqStart)
MADH2	Proprietary sequence (Sigma KiCqStart)
MADH3	Proprietary sequence (Sigma KiCqStart)
MADH4	Proprietary sequence (Sigma KiCqStart)
MADH7	Proprietary sequence (Qiagen QuantiTect)
TBR-I-Fwd	TGTTGGTACCCAAGGAAAGC
TBR-I-Rev	CACTCTGTGGTTTGGAGCAA
TBR-II-Fwd	ATGAGCAACTGCAGCATCAC
TBR-II-Rev	GGAGAAGCAGCATCTTCCAG
TGFB1	Proprietary sequence (Qiagen QuantiTect)
TGFB2	Proprietary sequence (Qiagen QuantiTect)
TGFB3	Proprietary sequence (Qiagen QuantiTect)
IL1B	Proprietary sequence (Qiagen QuantiTect)
IL6	Proprietary sequence (Qiagen QuantiTect)
TNF	Proprietary sequence (Qiagen QuantiTect)

Table S2. Apoptosis assessment. HaCaT cells were maintained either in full medium (FM) or serum deprived conditions (SS), in the absence or presence of TGF-β1 for the indicated time. Data represent percentage of combined early and late apoptotic cell fractions from three independent experiments ± SEM.

48 h	Control	TGF-β1
**FM**	3.2 ± 1.1%	5.1 ± 1.3%
**SS**	6.9 ± 1.8%	6.3 ± 1.6%

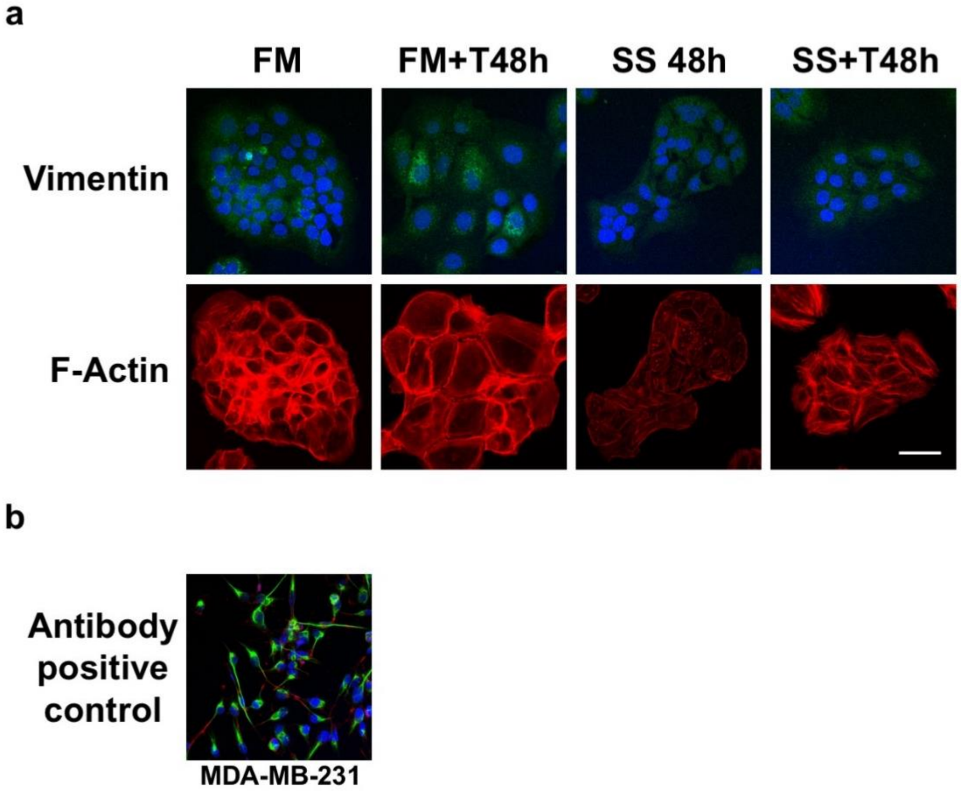

Figure S1. Continuous TGF-β1 stimulation does not induce Vimentin filaments in HaCaT cells**. (a)** Confocal microscopy studies on the expression and localization of Vimentin with or without TGF-β1. **(b)** Note the absence of Vimentin filaments when compared with positive controls performed on MDA-MB-231 breast cancer cell line. Representative images of at least three independent experiments are shown. Scale bar: 50 μm.

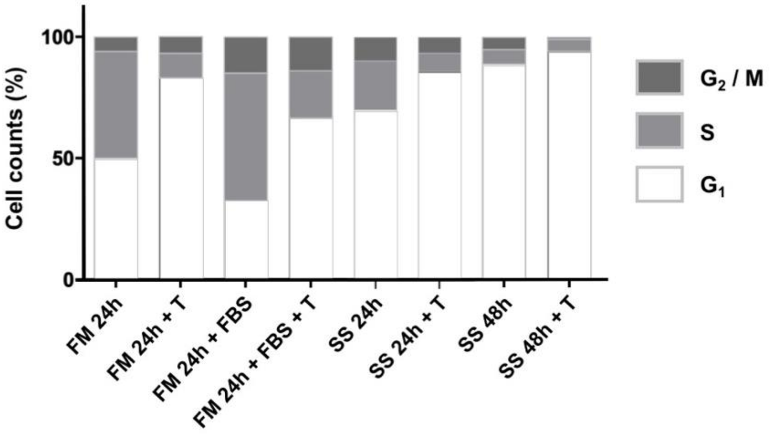

Figure S2. Acute TGF-β1 treatment induces cell cycle arrest to HaCaT keratinocytes. Cells were maintained either in full medium (FM) or serum deprived conditions (SS) for the indicated times, then treatments in the form of TGF-β1 [+ T] inoculation and/or extra fetal bovine serum (FBS) supplementation were applied. Representative data from at least three independent experiment are shown.
